# Supplementary material for: Innovation and constraint leading to complex multicellularity in the Ascomycota
Source: Nat Commun. 2017 Feb 8;8:14444. doi: 10.1038/ncomms14444 (PMC5309816; doi:10.1038/ncomms14444)
Supplement: Supplementary Information — Supplementary Figures, Supplementary Tables, Supplementary Methods and Supplementary References [file ncomms14444-s1.pdf]

Supplementary Figures

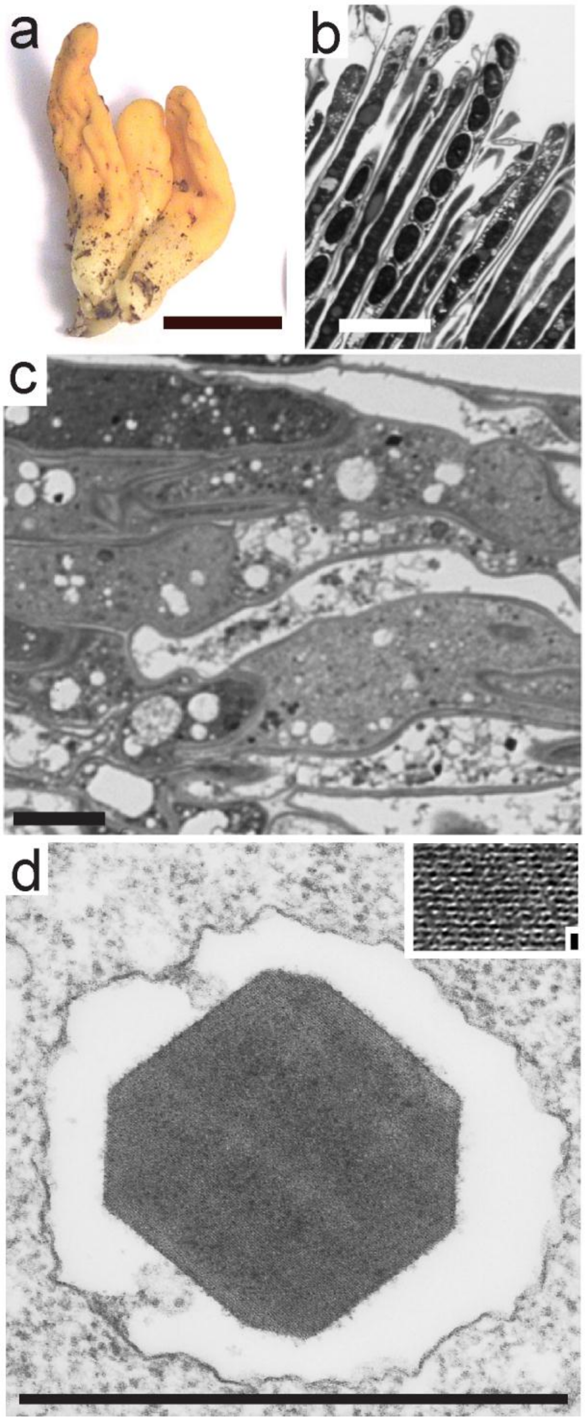

Supplementary Figure 1. *Neolecta* fruiting body structure. (a) *N. irregularis* fruiting bodies used for EM. Scale bar, 1 cm. (b) Thin section showing asci arrayed on fruiting body surface. Scale bar, 10 µm. (c) Thin section showing hyphal aggregates found in the core of the fruiting body. Scale bar, 10 µm. (d) Electron micrograph showing an octahedral crystal in the *N. irregularis* vacuole. Scale bar, 1 µm. The inset shows striations seen within the crystal lattice. Scale bar, 20 nm. This figure complements Fig. 1.

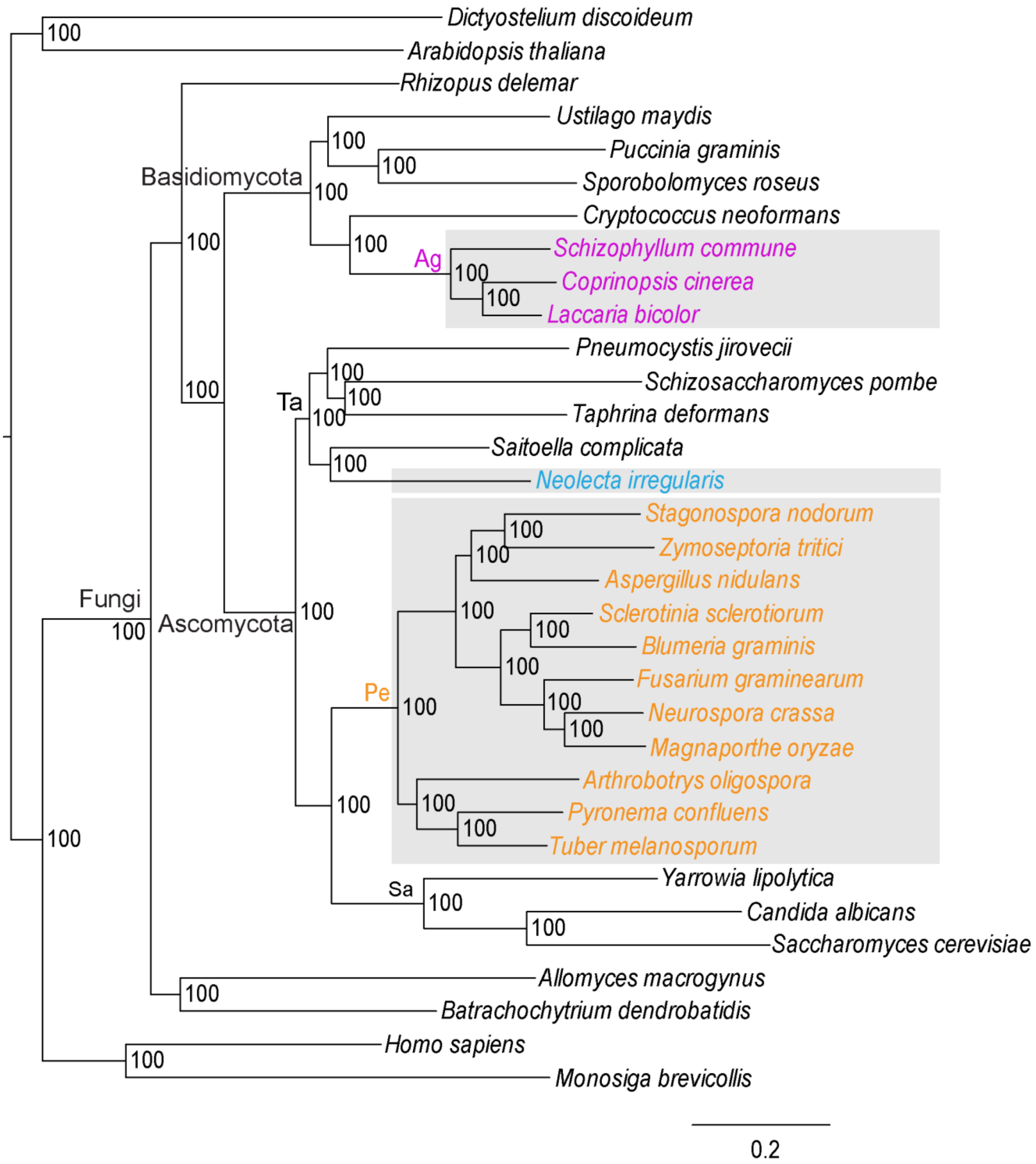

Supplementary Figure 2. Maximum likelihood phylogeny of fungi constructed using 110 single copy orthologs. Bootstrap support values are shown at the nodes. CM taxa are shown with grey background. Species coloring and abbreviations are as indicated in Fig. 1.

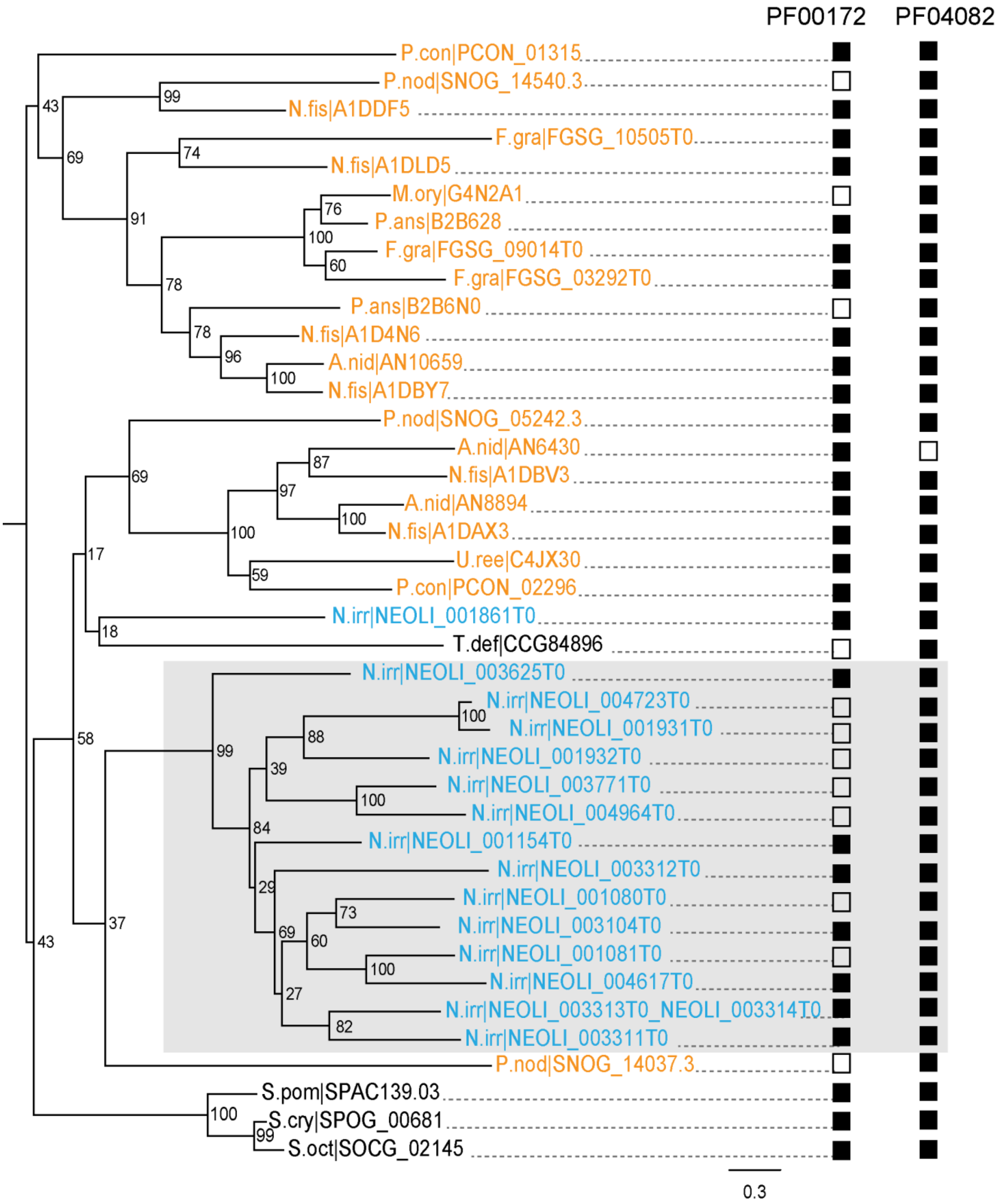

Supplementary Figure 3. Expansion of a fungal-specific transcription factor subfamily in *Neolecta*. *Neolecta* copies that appear to have undergone lineage-specific duplication are shown with grey background. The presence/absence of the two transcription factor domains: PF00172 (fungal Zn(2)-Cys(6) binuclear cluster domain) and PF04082 (fungal-specific transcription factor domain) is shown on the right. Filled squares indicate presence, empty squares indicate absence. Species coloring and abbreviations are as indicated in Fig. 1.

Supplementary Figure 4a

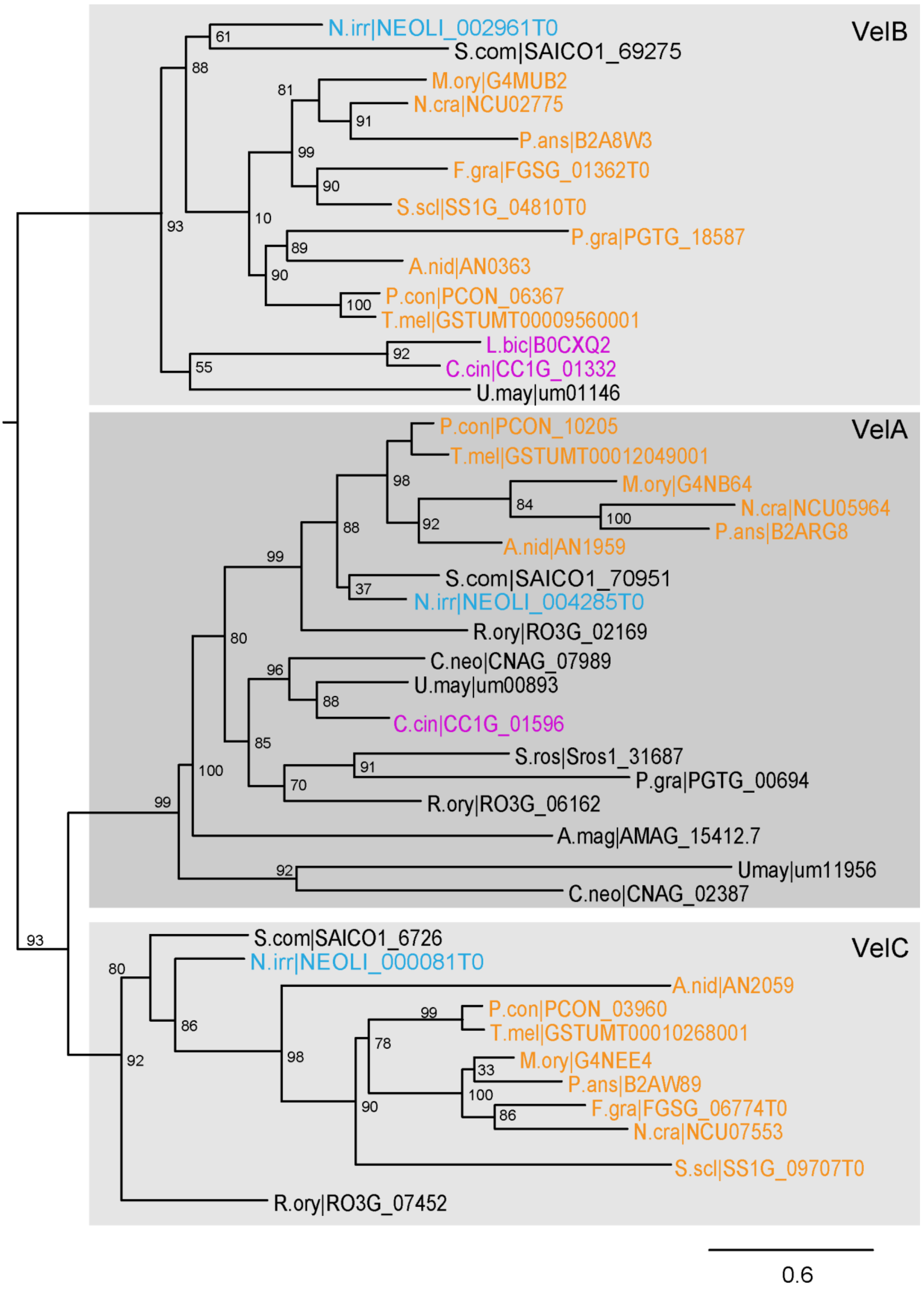

Supplementary Figure 4b

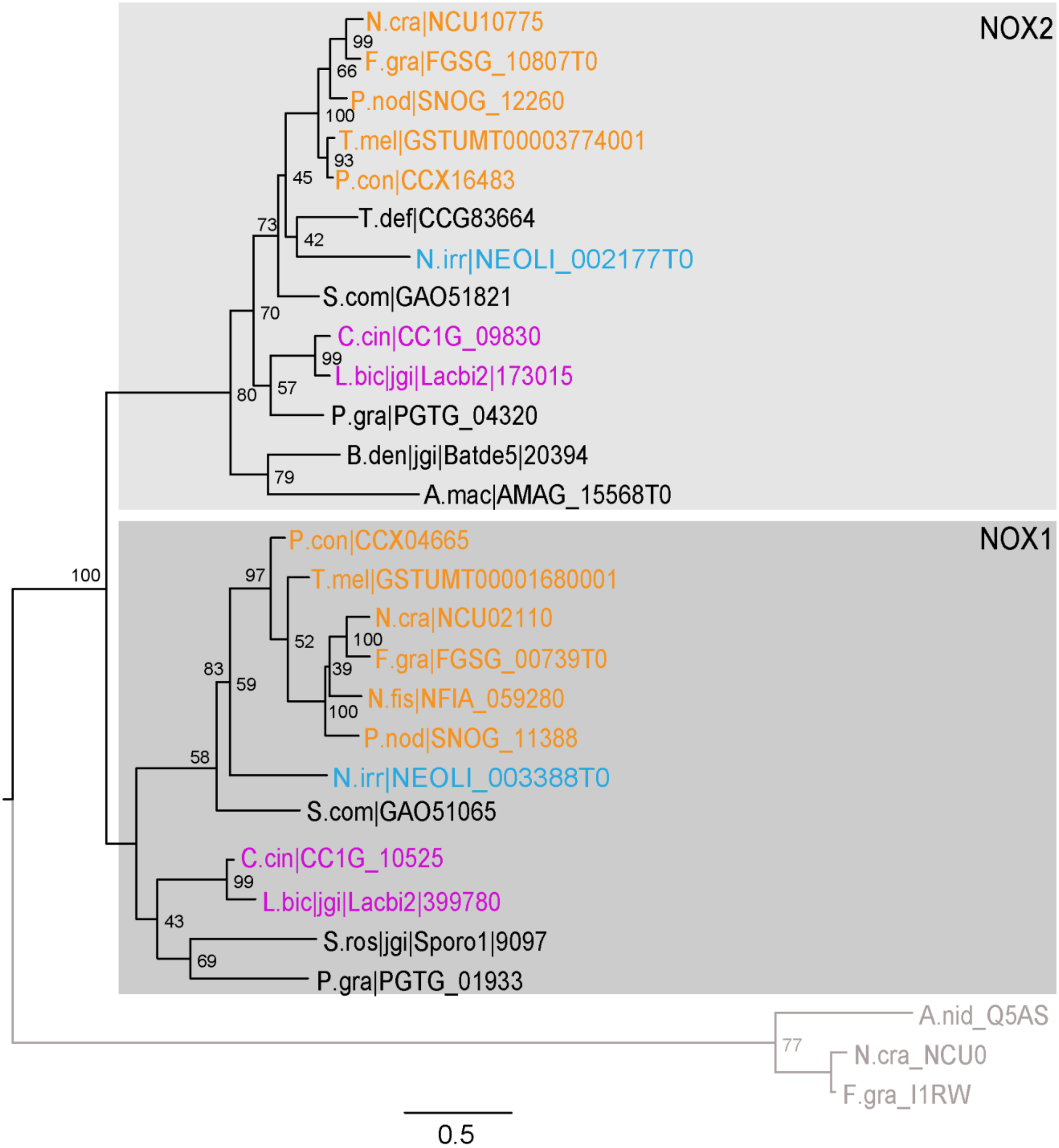

Supplementary Figure 4c

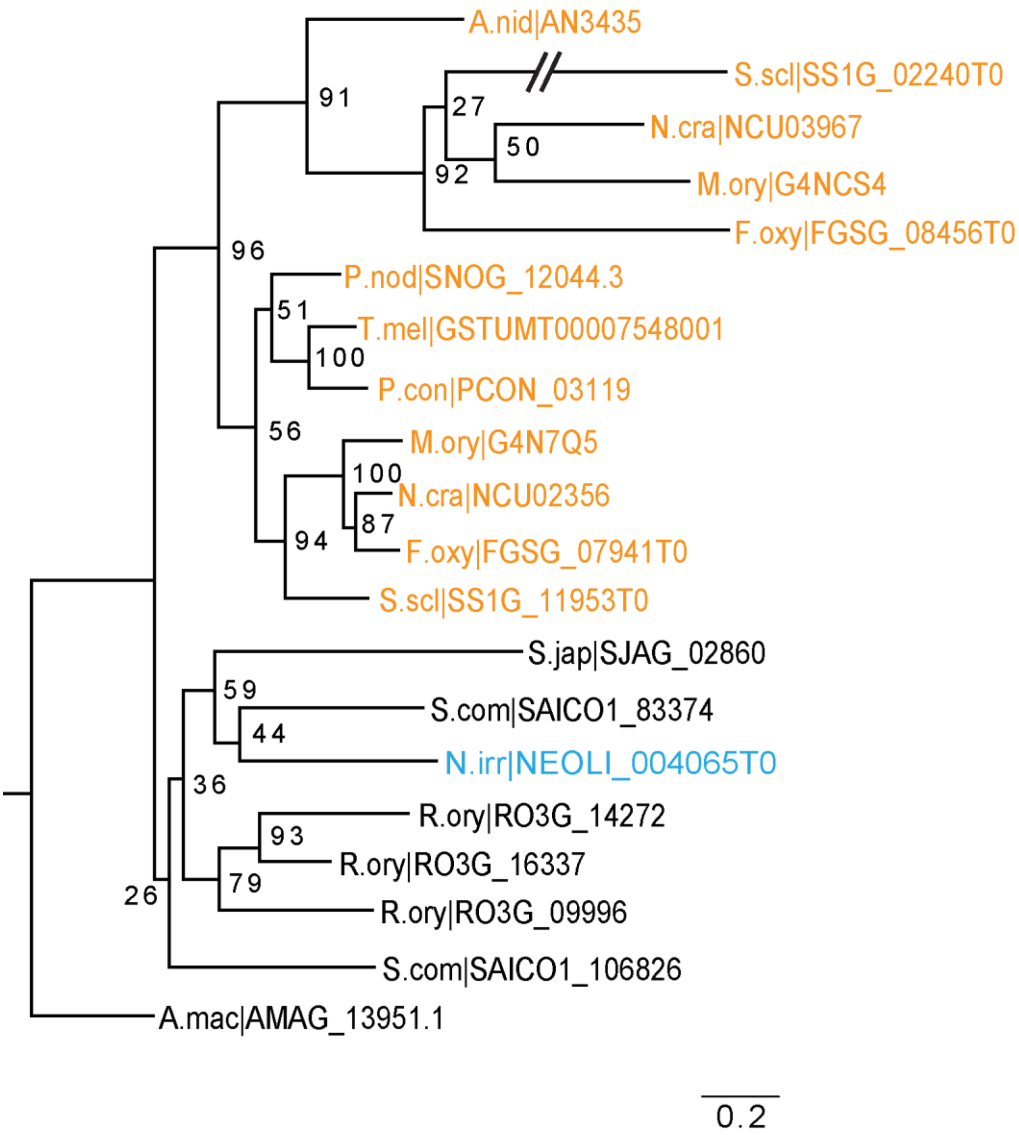

Supplementary Figure 4d

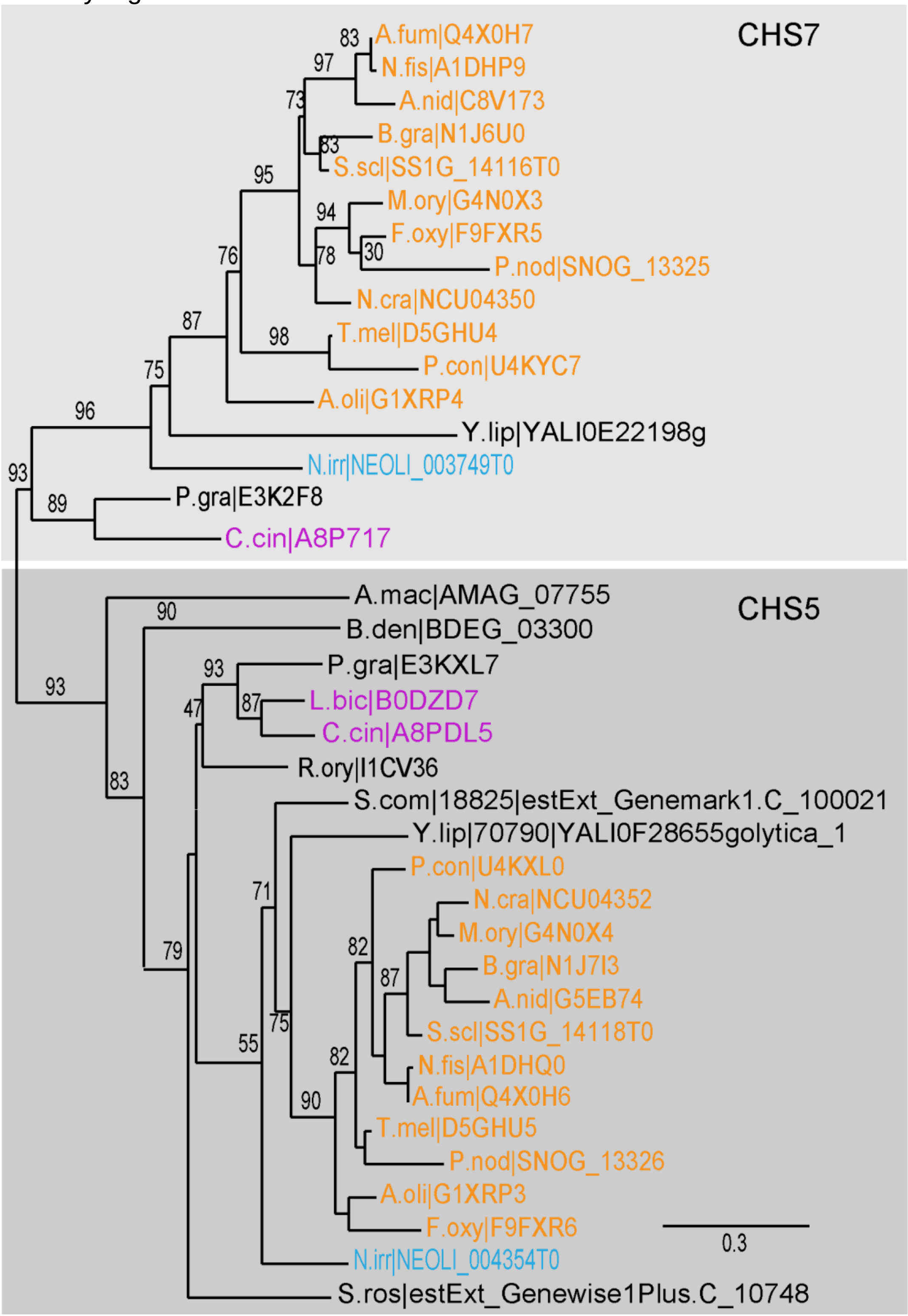

Supplementary Figure 4. Vertical transmission of known CM-associated genes. (a) Maximum likelihood tree of the Velvet family. (b) Maximum likelihood tree of NOX1/NOX2 homologs. The tree is rooted with Pezizomycotina ferric reductase sequences, which are highly related to the NOX family. These sequences and branches leading to them are shown in grey. (c) Maximum likelihood tree of WC1 homologs. (d) Maximum likelihood tree of CHS-7 and CHS-5 homologs. Nonparametric bootstrap support values are shown at the corresponding branches. Species coloring and abbreviations are as indicated in Fig. 1.

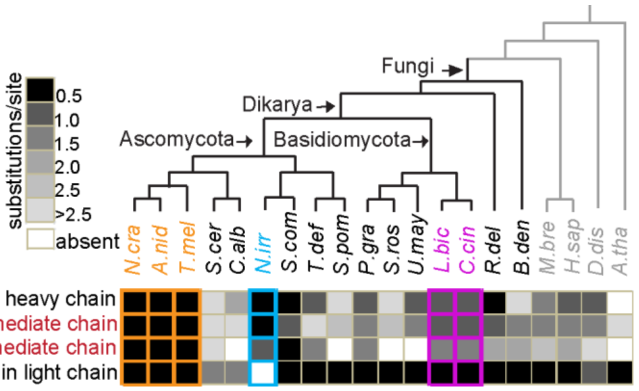

Supplementary Figure 5. Substitution rate analysis of dynein complex components. The degree of sequence divergence compared to the Pezizomycotina is determined by substitution rate and indicated by the grayscale. A lighter shade indicates greater divergence from the Pezizomycotina. Red text indicates components identified by our search for CM-associated proteins. The identification of dynein complex component orthologs is complicated by the presence of multiple highly conserved homologs of each component within a species. For a detailed description of the steps used to identify the orthologs, refer to Supplementary Methods. This figure complements Fig. 5.

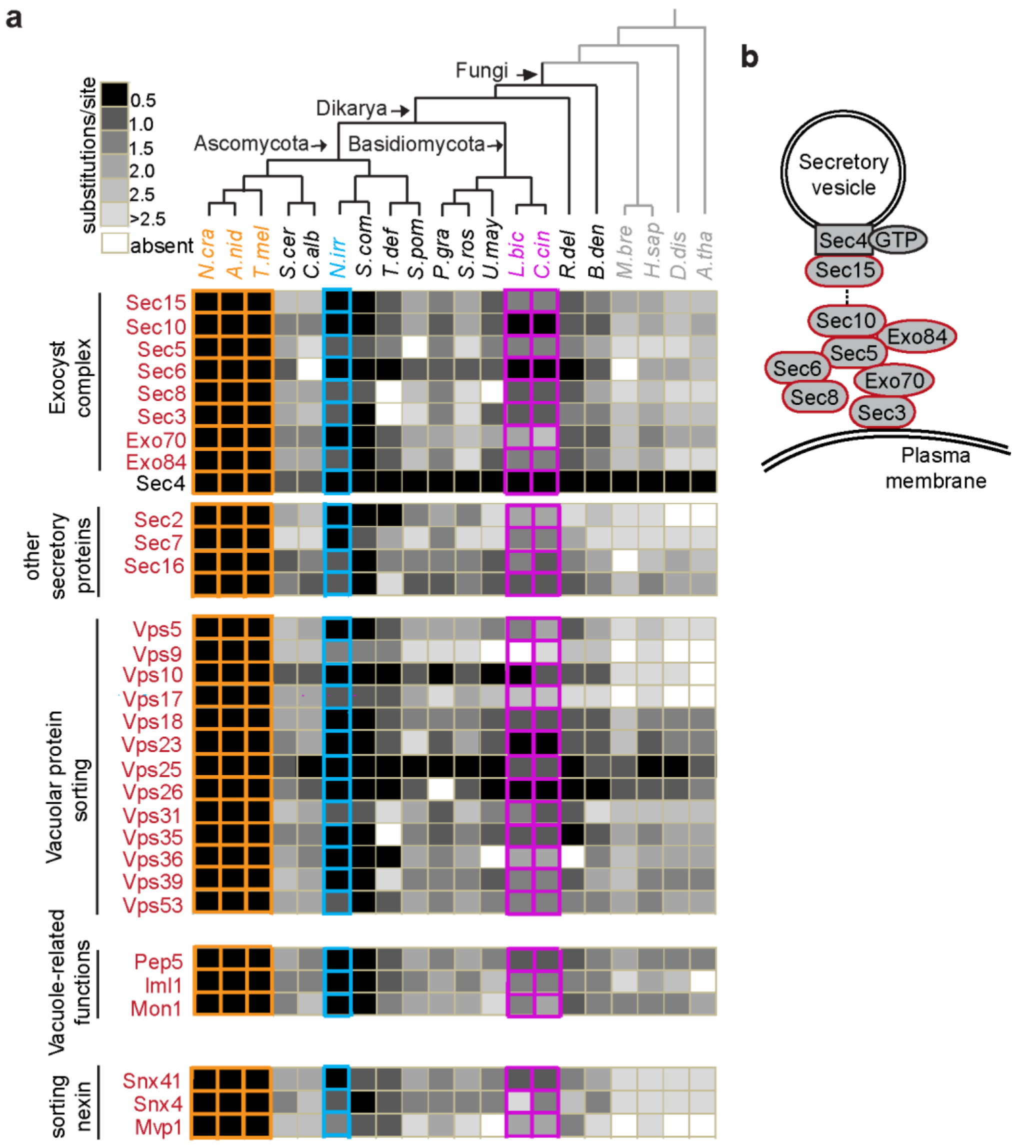

Supplementary Figure 6. Conservation and divergence of complexes involved in endomembrane organization. (a) Substitution rate analysis suggests a CM-associated constraint on the evolution of the exocyst complexes, vacuolar protein sorting functions, and other proteins involved in endomembrane organization. (b) The cartoon depicts the exocyst complex. All eight components of this complex were identified by the search for CM-associated sequences, as indicated by the red outline.

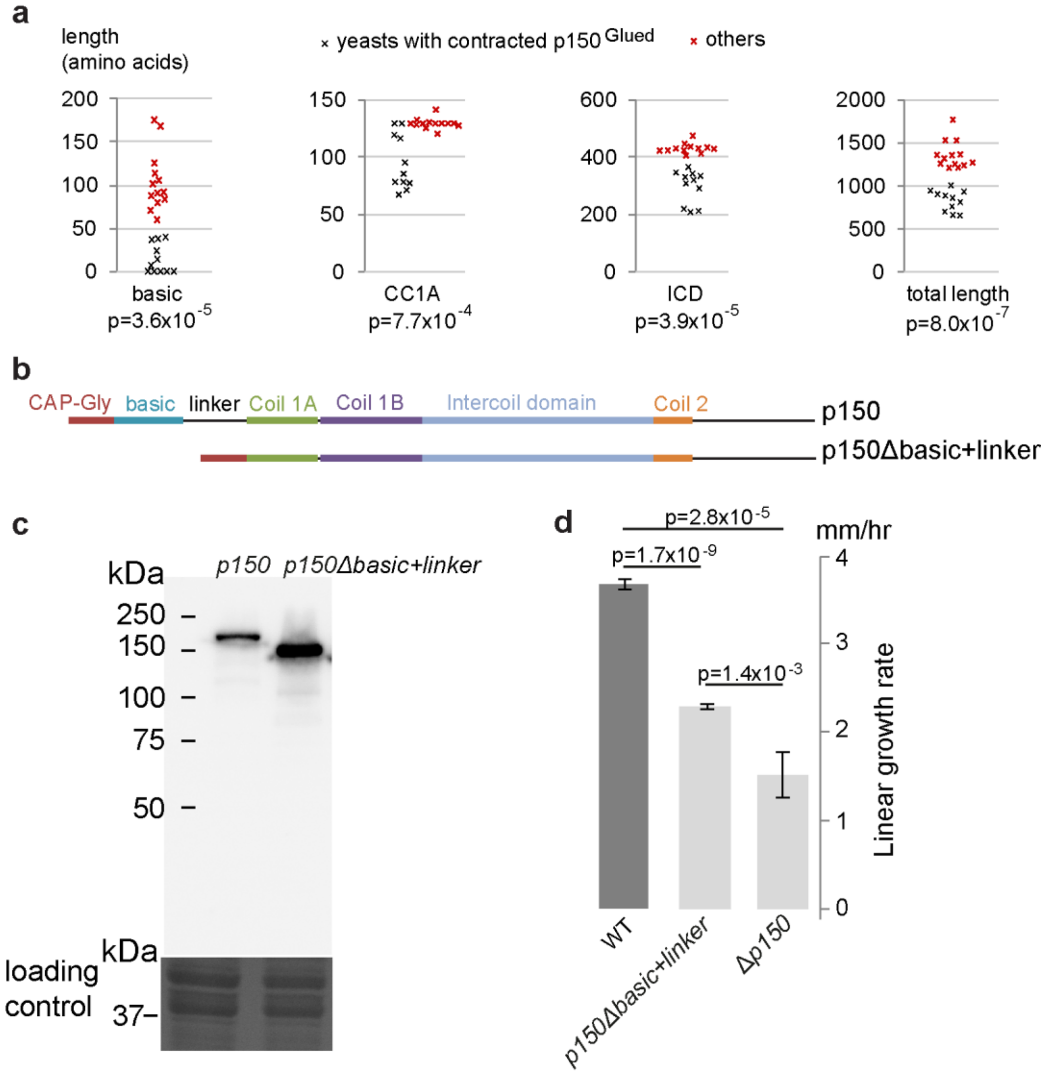

Supplementary Figure 7. p150^{Glued} domain length and the functional importance of the contracted basic domain and linker region. (a) Differences in domain length and total length between the group of yeasts highlighted with grey background in Fig. 5d and other species shown in Fig. 5d. Mann-Whitney U test p-values are shown below the indicated domains. Domain names shown below the graphs. (b) p150Glued domains in wild-type and variant p150^{Glued} without the basic and linker domains. (c) Steady-state levels of p150^{Glued} determined by western blotting (upper panel). The lower panel shows coomassie stained bands serving as a loading control. (d) Mean growth rate of wild-type and p150^{Glued} mutant strains. Error bars, s.d. (n=5). One-tailed t-test p-values are shown in the graph.

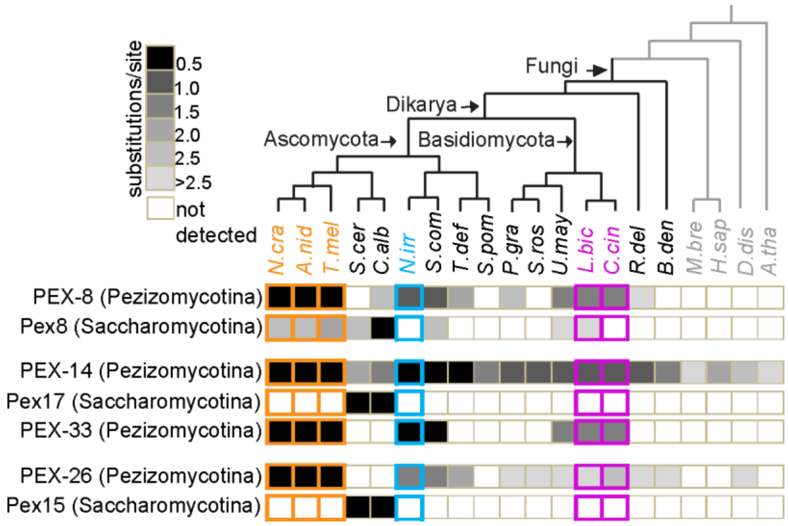

Supplementary Figure 8. Phylogenetic distribution of PEX-8, PEX-33, PEX-26 and their putative functional homologs in budding yeast. Search queries are from the clade shown in brackets.

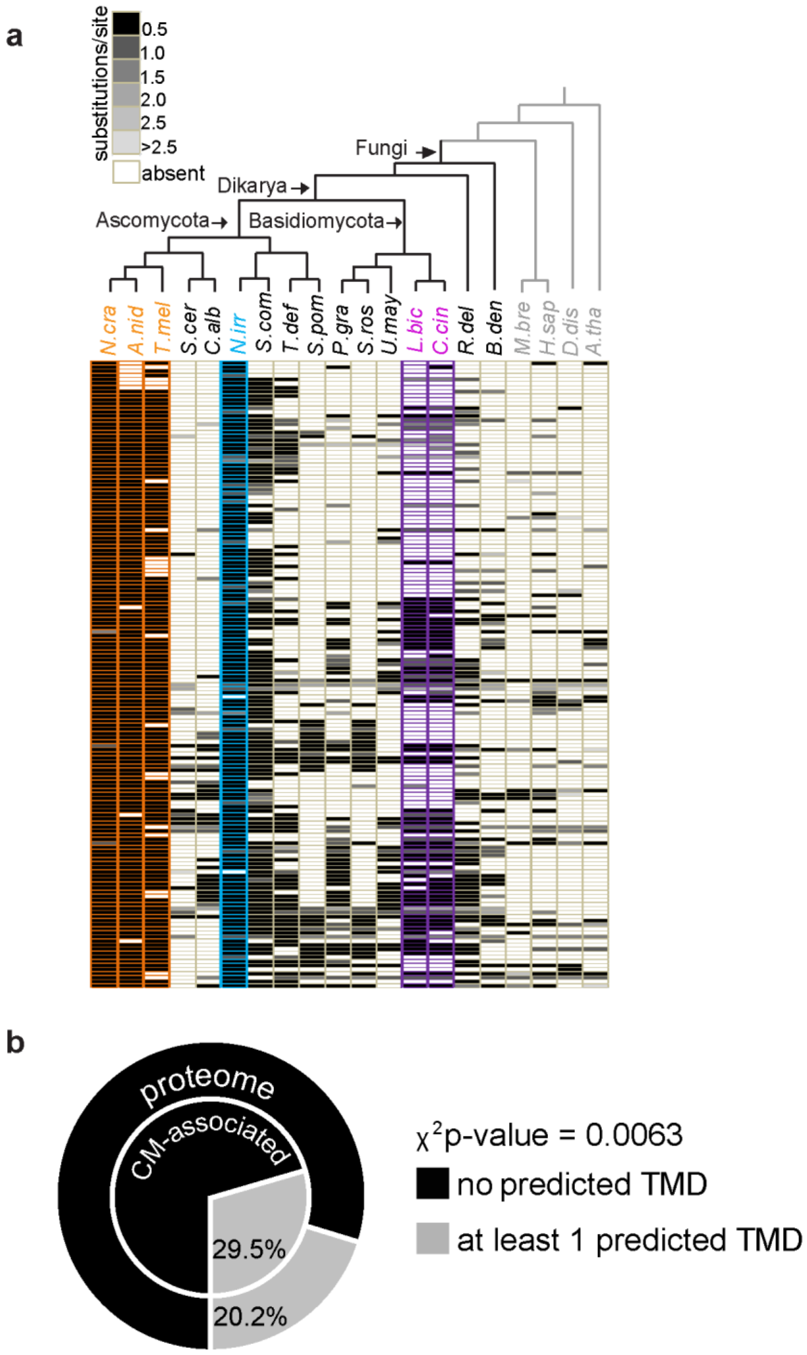

Supplementary Figure 9. Bioinformatic analyses of functionally characterized CM-associated genes. (a) Phylogenetic distribution of CM-associated genes functionally characterized. (b) Enrichment of proteins containing predicted transmembrane domain(s) in the group of CM-associated proteins.

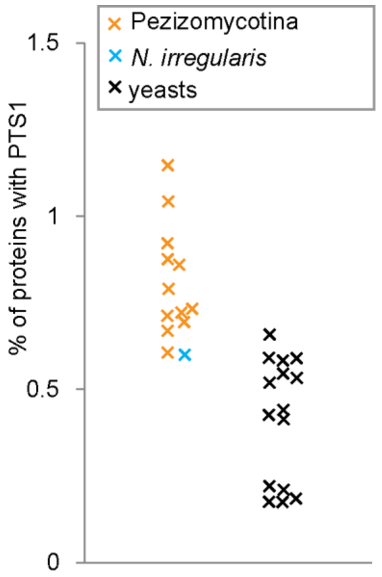

Supplementary Figure 10. Difference in percentage of predicted PTS1-bearing proteins in yeast and CM fungi proteomes in the Ascomycota. Mann-Whitney U test p-value = $2.1x10^{-7}$. Data used to generate this figure are summarized in Supplementary Table 4.

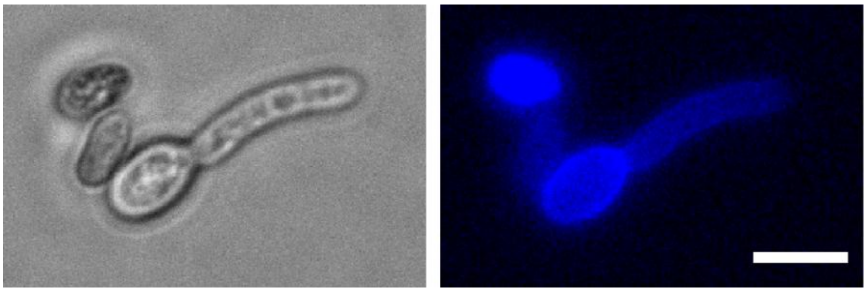

Supplementary Figure 11. Hypha-like growth in *Saitoella complicata* grown under nitrogen starvation. Left: Brightfield image, right: cell wall staining with Calcofluor. Scale bar, 10μm.

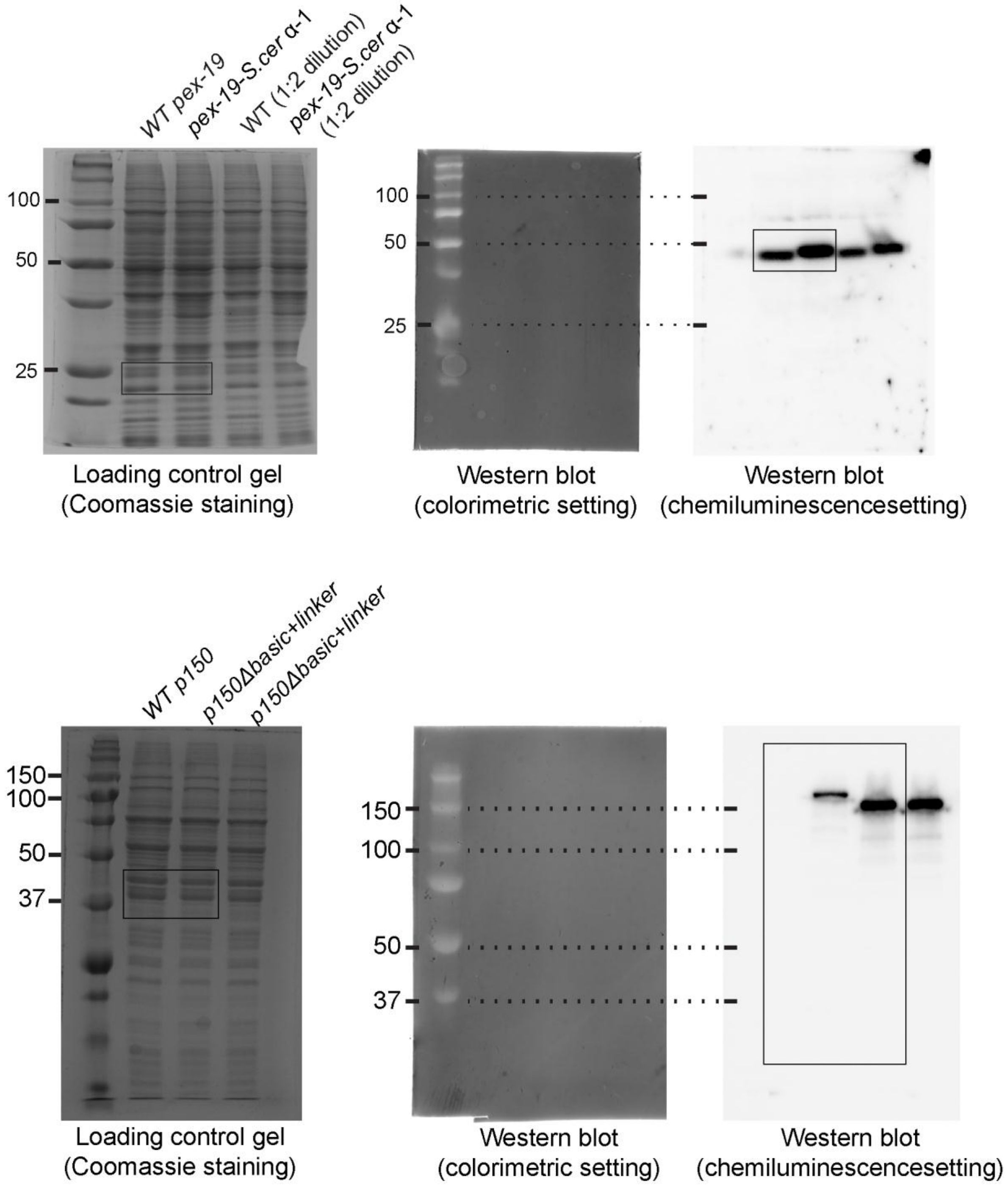

Supplementary Figure 12. Uncropped images of gels and blots shown in Fig. 6 (upper panel) and Supplementary Fig. 8 (lower panel). Sizes of molecular markers are shown in kDa. Boxed area indicates the portion of the gel/blot shown in the corresponding figure.

Supplementary Tables

Supplementary Table 1. Summary statistics of the *Neolecta irregularis* assembly

Genome size	14.5 Mb
Fold coverage	199
Number of contigs	7101
Number of RNAseq-based trancripts	24628 (97.1% aligned to assembly)
Repeated elements	12.8% of genome
Number of telomeric regions	10
Gene number	5546
Average gene size	1617 bp
Average intron density	4.3 introns per gene
Average intron length	42 bp
Gene density	2.6 kb per gene

Supplementary Table 2. Enriched functional categories in the set of CM-associated proteins

FunCat ID	Category	χ2 p-value
20.01	substrate transport	2.68E-05
20.09	transport routes	2.69E-03
34.11.12	nutrient perception and nutritional adaptation	3.01E-04
1.2	secondary metabolism	3.78E-04
1.6	lipid, fatty acid and isoprenoid metabolism	1.28E-06
42.19	peroxisome biogenesis	1.18E-03
41.01.03	tissue patterning	1.93E-03
2.11	electron transport and membrane-associated energy conservation	2.22E-03
16.21.05	FAD/FMN binding	1.81E-09
2.13.03	aerobic respiration	8.17E-04
32.07.01	detoxification involving cytochrome P450	1.12E-07
32.10.07	degradation/modification of exogenous polysaccharides	5.27E-04
34.01.03	anion homeostasis	4.29E-03

Supplementary Table 3. Proteomes used in phylogenetic analyses

Species	Taxon	Source
Neurospora crassa	Pezizomycotina (Ascomycota, CM)	[1]
Sordaria macrospora		[1]
Magnaporthe oryzae		[2]
Fusarium oxysporum		[3]
Blumeria graminis		[4]
Botryotinia fuckeliana		[5]
Sclerotinia sclerotiorum		[1]
Neosartorya fischeri		[6]
Aspergillus fumigatus		[7]
Aspergillus nidulans		[8]
Phaeosphaeria nodorum		[1]
Arthrobotrys oligospora		[9]
Pyronema confluens		[10]
Tuber melanosporum		[11]
Candida albicans	Saccharomycotina (Ascomycota, non-CM)	[1]
Candida dubliniensis		[12]
Candida glabrata		[13]
Candida parapsilosis		[14]
Saccharomyces cerevisiae		[15]
Ashbya gossypii		[16]
Yarrowia lipolytica		[13]
Lachancea thermotolerans		[17]
Zygosaccharomyces rouxii		[17]
Kluyveromyces dobzhanskii		[13]
Kazachstania naganishii		[18]
Tetrapisispora phaffii		[18]

(Supplementary Table 3, continued)

Species	Taxon	Source
Taphrina deformans	Taphrinomycotina (Ascomycota, non-CM)	[19]
Schizosaccharomyces pombe		[20]
Schizosaccharomyces cryophilus		[21]
Schizosaccharomyces japonicus		[21]
Schizosaccharomyces octosporus		[21]
Pneumocystis jirovecii		[22]
Saitoella complicata		[23]
Sporobolomyces roseus	Basidiomycota (non-CM)	[23]
Ustilago maydis		[24]
Cryptococcus neoformans		[25]
Puccinia graminis		[26]
Coprinopsis cinerea	Agaricomycotina (Basidiomycota, CM)	[27]
Laccaria bicolor		[28]
Allomyces macrogynus	Fungi (early diverging)	[1]
Batrachochytrium dendrobatidis		[23]
Rhizopus oryzae		[29]
Dictyostelium discoideum	Opisthokonta	[30]
Monosiga brevicollis		[31]
Salpingoeca rosetta		[32]
Homo sapiens		[33]
Arabidopsis thaliana	Plant	[34]

Supplementary Table 4. Percentage of predicted PTS1-bearing proteins in yeast and CM species' proteomes

	Species	Predicted proteome size	Number of predicted PTS1-bearing proteins	Percentage of predicted PTS1-bearing proteins (%)
Pezizomycotina	*M.oryzae*	12647	90	0.71
	F.oxysporum	17784	164	0.92
	S.sclerotiorum	14503	97	0.67
	B.fuckeliana	16338	99	0.61
	N.fischeri	10392	91	0.88
	A.nidulans	10555	121	1.15
	P.confluens	13306	96	0.72
	T.melanosporum	7494	52	0.69
	A.oligospora	11478	84	0.73
	P.nodorum	12379	129	1.04
	A.fumigatus	9649	83	0.86
	N.crassa	10258	81	0.79
	N.irregularis	6674	40	0.60
Ascomycota yeasts	*S.cerevisiae*	6575	28	0.43
	C.albicans	5931	35	0.59
	S.pombe	5144	9	0.17
	S.cryophilus	5180	9	0.17
	S.japonicus	4878	9	0.18
	S.octosporus	4986	11	0.22
	C.dubliniensis	5829	34	0.58
	C.glabrata	5200	27	0.52
	C.parapsilosis	5777	38	0.66
	A.gossypii	4768	26	0.55
	L.thermotolerans	5092	30	0.59
	Z.rouxii	4991	22	0.44
	K.dobzhanskii	5076	27	0.53
	K.naganishii	5321	22	0.41
	T.phaffii	5253	11	0.21

Supplementary Table 5. Strains and primers

Strain ID	Genotype and primers	Source
GSF3256	Δ*pex-19*::*hph*, *csr-1::phex-mCherry-pts1*	[35]
GSF3839	Δ*pex-19*::*hph*, *his-3* allele 1-234-723::*pex-19*, *csr-1::phex-mCherry-pts1*	[35]
GSF4397	Δ*pex-19*::*hph*, *his-3* allele 1-234-723::*pex-19Scer*α*-1*, *csr-1::phex-mCherry-pts1*<br><br>Primers:<br>pex19F: CAGATTACGCTCATAGCCATATGGCTTCGAATAACGCC<br>pex19R: GGATGTGTTGAGACAAGGATCCTTACTGAGGGGCGCAT<br>Scer-α1F:<br>ATGGACGACGCGATTACGAAAATCCTGGACCAGATGACACACAAGGACATTCTGTA<br>*Scer*-α1R:<br>CATCTGGTCCAGGATTTTCGTAATCGCGTCGTCCATCTCCTCTTCGCTGCCCTCG	This study
GSF4402	*hep-1::hep-1-hph-gfp*<br><br>Primers:<br>Primer 1: TTTCCTCAGCACCCTCACTC<br>Primer 2: GTGAGTTCAGGCTTTTTCATCATTTTGAAGAGTTGTGGTAGGGGAA<br>Primer 3: TTCCCCTACCACAACTCTTCAAAATGATGAAAAAGCCTGAACTCAC<br>Primer 4: TGGGCGACTTACGGTTGTCGTGCTTGTACAGCTCGTCCATGC<br>Primer 5: GCATGGACGAGCTGTACAAGCACGACAACCGTAAGTCGCCCA<br>Primer 6: TGAGTCAACCCAGCGATACT<br>Primer 7: ACTGGGAGTTGACGTAGAGA<br>Primer 8: ATCTGGCACCGCCATCTCACTT	This study
GSF4403	*hep-2::hph-gfp-hep-2*<br><br>Primers:<br>Primer 1: CCCAAACGTCCCCATAGAGT<br>Primer 2: CGGTGAGTTCAGGCTTTTTCATACGGTACTCCTTCTCCTTGAGG<br>Primer 3: CCTCAAGGAGAAGGAGTACCGTATGAAAAAGCCTGAACTCACCG<br>Primer 4: TTGTGCCGTTTATATCTACACTTTACTTGTACAGCTCGTCCATGC<br>Primer 5: GCATGGACGAGCTGTACAAGTAAAGTGTAGATATAAACGGCACAA<br>Primer 6: CATGGCTGCCGTTTTGTGA<br>Primer 7: CCCAAACGTCCCCATAGAGT<br>Primer 8: CATGGCTGCCGTTTTGTGA	This study
GSF4400	*hep-3::hep-3-hph-gfp*<br><br>Primers:<br>Primer 1: GATGAGCGGTGACTACGACA<br>Primer 2: GCGGTGAGTTCAGGCTTTTTCATTTTGTCAAGGTTCTT<br>Primer 3: AAGAACCTTGACAAAATGAAAAAGCCTGAACTCACCGC<br>Primer 4: TTCCACTTTGGTAGGCACCTTGTACAGCTCGTCCATGCCGA<br>Primer 5: TCGGCATGGACGAGCTGTACAAGGTGCCTACCAAAGTGGAA<br>Primer 6: CCACCCACCTAGTCCCT<br>Primer 7: GAGCTGATGCAGAAGACCGA<br>Primer 8: TATCTGATGACACCGTCGCC	This study
GSF4399	*spz-1::spz-1-hph-gfp*<br><br>Primers:<br>Primer 1: CCATCAACGCTGCACTCCG<br>Primer 2: GCGGTGAGTTCAGGCTTTTTAGCCCTGACTTCCGTAGC<br>Primer 3: GCTACGGAAGTCAGGGCTAAAAAGCCTGAACTCACCGC<br>Primer 4: AACAAGTTCAGTCTCCTACTTGTACAGCTCGTCCATGCCGA<br>Primer 5: TCGGCATGGACGAGCTGTACAAGTAGGAGACTGAACTTGTT<br>Primer 6: TGGGTTAGGGATTTAGAGTTGT<br>Primer 7: TGAGCAACTGTCTGACTCGG<br>Primer 8: GTGCGAGCGCTTTACTATTGG	This study

(Supplementary Table 5, continued)

Strain ID	Genotype and primers	Source
GSF4401	*rogdi::rogdi-hph-gfp*<br><br>Primers:<br>Primer 1: CGGAACACGGATAGTCAAGGG<br>Primer 2: GCGGTGAGTTCAGGCTTTTTATCCTCCAGCTCCTCTCC<br>Primer 3: GGAGAGGAGCTGGAGGATAAAAAGCCTGAACTCACCGC<br>Primer 4: CAGCCGTCGGGCCCTCTACTTGTACAGCTCGTCCATGCCGA<br>Primer 5: TCGGCATGGACGAGCTGTACAAGTAGAGGGCCCGACGGCTG<br>Primer 6: CCTCCTTTCCAAGCGACA<br>Primer 7: TACTCAGCACACCACGGAAC<br>Primer 8: TGGGAATACCAGCGCTACAC	This study
GSF4404	*vezatin::vezatin-hph-gfp*<br><br>Primers:<br>Primer 1: TCATCCCGTATTCTTGCACA<br>Primer 2: GGTGAGTTCAGGCTTTTTCATTAAAGAAATCCTCGTCGGC<br>Primer 3: GCCGACGAGGATTTCTTTAATGAAAAAGCCTGAACTCACC<br>Primer 4: GCAGAGTTTGTCTGTCGTCACTTGTACAGCTCGTCCATGC<br>Primer 5: GCATGGACGAGCTGTACAAGTGACGACAGACAAACTCTGC<br>Primer 6: GGAACCACCAATACCACAGG<br>Primer 7: CGAGAGGCTTCGTCTCATCT<br>Primer 8: GCTCGTGGTTTGACGAAGA	This study
GSF4405	*mit-1::hph-gfp-mit-1*<br><br>Primers:<br>Primer 1: TAGCTCTTCGGACACCTTGG<br>Primer 2: GGTGAGTTCAGGCTTTTTCATCATGTCGACCAGTATCTATCG<br>Primer 3: CGATAGATACTGGTCGACATGATGAAAAAGCCTGAACTCACC<br>Primer 4: GGATTGGACTTGTATCGTCTCTTGTACAGCTCGTCCATGC<br>Primer 5: GCATGGACGAGCTGTACAAGAGACGATACAAGTCCAATCC<br>Primer 6: GACCTTGAATAACCGTTGCG<br>Primer 7: CGACGCTGAATGTAAGACCG<br>Primer 8: CGTGCAGAATTGAGGGTCTT	This study
GSF4407	*p150Glued::hph-HA-p150GluedΔbasic+linker*<br><br>Primers:<br>Primer 1: AGGTGCGACTTTGGAGCTAA<br>Primer 2: GCTCCTTCAATATCATCTTGCGTGTACTATCTCCATTGCTTCATGACG<br>Primer 3: CGTCATGAAGCAATGGAGATAGTACACGCAAGATGATATTGAAGGAGC<br>Primer 4: CCATACTCCCAGGGTATCTGCTACTTTTAATTAAGTTCGGTCGGCATC<br>Primer 5: GATGCCGACCGAACTTAATTAAAAGTAGCAGATACCCTGGGAGTATGG<br>Primer 6: GAACATCGTAAGGGTAGCTGCCCATGTTGTTGGGTTGCGAG<br>Primer 7: ACATGGGCAGCTACCCTTACGATGTTCCTGACTATGCGTCCGAGCTAGGAGTAGCAGTAGG<br>Primer 8: TGACCCTCGTGATGCTCGTCTGGGCAATGACATTGAAGG<br>Primer 9: CCTTCAATGTCATTGCCCAGACGAGCATCACGAGGGTCA<br>Primer 10: CCTTGTTCATAGTCGCAGTATTCGTAAGCTGTTTCGTAGGTGACCTCG<br>Primer 11: CGAGGTCACCTACGAAACAGCTTACGAATACTGCGACTATGAACAAGG<br>Primer 12: TGATCTCCTCGGATTGGTTC	This study

(Supplementary Table 5, continued)

Strain ID	Genotype and primers	Source
GSF4504	*p150Glued::hph-HA-p150Glued*<br><br>Primers:<br>Primer 1: CCTTGGAGAGCGACAGAGAC<br>Primer 2: GAACATCGTAAGGGTATGCCATAACAACAACCGCCAACCTC<br>Primer 3: GTTGTTGGCAGCTACCCTTACGATGTTCCTGACTATGCGTGATGATGG GAAGTGATGATGA<br>Primer 4: GCTCCTTCAATATCATCTTGCGGCTCGCCTTTCGGTGAAC<br>Primer 5: GTTCACCGAAAGGCGAGCCGCAAGATGATATTGAAGGAGC<br>Primer 6: GTGGTCCGTGCACCGAATTAATTAAGTTCGGTCGGCATC<br>Primer 7: GATGCCGACCGAACTTAATTAATTCGGTGCACGGACCAC<br>Primer 8: GAGCACCTCGGTCTTCTTGAC<br>Primer 9: GAGACGCTCGAGTCTCGTATG<br>Primer 10: CGTACACCTCCTTGGTCTCG	This study

Supplementary Table 6. Protein names and references

Figure	Protein name	Reference(s)
3a	HEX	36
	WSC	37
	LEASHIN	38
	SPA-9, SPA-1, SPA-18	39
	SPC-33,SPC-14	40
3b	HAM-11	41
	HAM-5	42
	HAM-8	43
	SOFT (also known as PRO40)	44,45
	HAM-7	43,46
	PRO1	47
	PRO41<br>(also known as HAM-6)	43,48,49
	HAM-9	43
	GAT1, BRI1	50
	PRO44	10
	ADA1	51,52
	NRC-1	53,54
	HAM-10	43
	CFS1	55
	RCO-1	42
	SNF-5, HAM-1	43
	RAC-1	43,56
	MAK-2	57–59
	MIK-1, MAK-1, MEK-1	58,60
	MOB3	61
	HAM-2 (PRO22)	62,63
	PRO11<br>(also known as HAM-3)	64–66
	ACL1	67
	HAM-4	65,68
	PP2A	43
	ASF1	69
4a	WC-1, WC-2	70
	ppoC	71
	velA, velB, velC, LaeA	72<br>73
	vosA	74<br>75
	NOX-1, NOX-2, NOX-R	76
	PRO41<br>(also known as HAM-6)	43,48,49
4b	SPA-10	39
	CHS-5, CHS-7	77,78

Supplementary Methods

Genome and transcriptome sequencing and assembly

Neolecta irregularis fruiting bodies were collected on Black Mountain, New Hampshire and stored at -80°C. Paired-end genomic DNA was sequenced using the TruSeq Illumina protocol (33.8 Gb of data, insert size = 300 bp). Reads were assembled using Velvet[79]. Total RNA was sequenced using Illumina paired-end technology (6.6 Gb of data). Transcriptome alignment and gene expression assessment were performed using Tophat[80] and Cufflinks[81]. *de novo* assembly was performed with Trinity[82]. Transcripts were aligned to the genome assembly using PASA[83].

Annotation and phylogeny

Full-length transcripts were selected from PASA output and used to train AUGUSTUS[84] and SNAP[85]. GenMark-ES was self-trained on genome sequence[86]. *Ab initio* predictions, homology and transcriptome based evidences were merged into gene models using MAKER[87].

To build the species tree, we extracted 110 single copy orthologs from OrthoMCL clustering. Each cluster was aligned independently using T-Coffee[88] and merged. Ambiguous regions were removed using Trimal[89] with automated option. The final matrix contains 33,355 sites and 2.18% of proportions of gaps and undetermined characters. The best-fit model was determined using RAxML[90] with ProteinModelSelection function. The maximum likelihood phylogeny was inferred using RAxML with the LG model[91] and 100 bootstrap replicates.

Gene family gain-loss and expansion analyses

Proteome clustering of 20 species (Supplementary Table 3) was performed with OrthoMCL v2[92] using 2.0 inflation parameter and ssearch36[93] (e-value = 1e^{-4}). Gene family gains and losses were projected onto species phylogeny using Dollo parsimony with Count[94]. As an unavoidable consequence of a limited number of species, proteins

with restricted taxonomic distribution which do not have detectable homologs in any other species included are not assigned to any families by OrthoMCL. These were mapped to the nodes where they are likely to have originated using their blast output against the nr database (BLASTP, e-value = $1e^{-15}$).

Gene family size variation was computed using CAFE3[95] (p-value = 0.05) to search for expanded gene families in *Neolecta*. Duplications of the fungus-specific transcription factor subfamily were analyzed by examining homologous sequences retrieved using phmmer (e-value = $1e^{-5}$) with NEOLI_001080T0 as query. Sequences were aligned with MCoffee[88]. The resulting alignment was trimmed using trimAl[89] with -automated1 option. The phylogenetic tree was generated using RAxML[90] v.8.1.20 with 100 bootstraps. The final alignment contains 242 alignment characters and 7.73% of proportions of gaps and undetermined characters. The tree was visualized using FigTree (http://tree.bio.ed.ac.uk/software/Figtree/).

Identification of dynein complex component orthologs

Components of the dynein complex are present in different copy numbers in different species, which makes it insufficient to identify orthologs by searching for reciprocal best BLAST hits. Because of the difficulties in identifying true orthologs caused by a high level of conservation among all copies of the same gene, we limited our analysis to the four main components of the dynein complex: heavy chain 1 (*DYNC1H1*, Q14204), intermediate chain 1 (*DYNC1I1*, O14576), light intermediate chain 1 (*DYNC1LI1*, Q9Y6G9), and light chain 1 (*DYNLL1*, P63167). *Homo sapiens* sequences were first queried against target proteomes using BLASTp. The top 4 hits from each target proteomes were extracted. These hits were used to build a multiple sequence alignment using MUSCLE[96], which was then trimmed using trimAl[89] (gap cutoff 0.8 and conservation cutoff 0.5) and used to construct a maximum likelihood tree using PhyML[97]. The hit from each target species that is most closely clustered with the initial *H. sapiens*

query sequence in the resulting tree was considered an ortholog of the query sequence, and included in our analysis shown in Supplementary Fig. 5.

Identification of candidate CM-associated genes

These examples illustrate the selection criteria of the search for candidate CM-associated genes described in the Methods.

Example	$-\log_{10}$(e-value)		Difference	Range of $-\log_{10}$(e-value of *N.irr*)	Selected?
	S.cer	*N.irr*			
1	30	50	20	[1,100)	yes
2	30	40	10		no
3	130	170	40	[100,200]	yes
4	130	140	10		no
5	230	290	60	(200,+∞)	yes
6	230	270	40		no

Supplementary References

1. Broad Institute. at <https://www.broadinstitute.org/>
2. Dean, R. A. *et al.* The genome sequence of the rice blast fungus *Magnaporthe grisea*. **434,** (2005).
3. Ma, L. J., van der Does, H. C., Borkovich, K. a & Al, E. Comparative analysis reveals mobile pathogenicity chromosomes in *Fusarium*. *Nature* **464,** 367–373 (2010).
4. Spanu, P. D. *et al.* Genome expansion and gene loss in powdery mildew fungi reveal tradeoffs in extreme parasitism. *Science.* **330,** 1543–1546 (2010).
5. Amselem, J. *et al.* Genomic analysis of the necrotrophic fungal pathogens *Sclerotinia sclerotiorum* and *Botrytis cinerea*. *PLoS Genet.* **7,** (2011).
6. Fedorova, N. D. *et al.* Genomic islands in the pathogenic filamentous fungus *Aspergillus fumigatus*. *PLoS Genet.* **4,** (2008).
7. Nierman, W. *et al.* Genomic sequence of the pathogenic and allergenic filamentous fungus *Aspergillus fumigatus. Nature* **438,** 1151–6 (2005).
8. Galagan, J. E. *et al.* Sequencing of *Aspergillus nidulans* and comparative analysis with *A. fumigatus* and *A. oryzae*. *Nature* **438,** 1105–1115 (2005).
9. Yang, J. *et al.* Genomic and proteomic analyses of the fungus *Arthrobotrys oligospora* provide insights into nematode-trap formation. *PLoS Pathog.* **7,** (2011).
10. Traeger, S. *et al.* The Genome and Development-Dependent Transcriptomes of *Pyronema confluens*: A Window into Fungal Evolution. *PLoS Genet.* **9,** e1003820 (2013).
11. Martin, F. *et al.* Périgord black truffle genome uncovers evolutionary origins and mechanisms of symbiosis. *Nature* **464,** 1033–8 (2010).
12. Jackson, A. P. *et al.* Comparative genomics of the fungal pathogens *Candida dubliniensis* and *Candida albicans*. *Genome Res.* **19,** 2231–2244 (2009).
13. Dujon, B. *et al.* Genome evolution in yeasts. *Nature* **430,** 35–44 (2004).
14. Butler, G. *et al.* Evolution of pathogenicity and sexual reproduction in eight *Candida* genomes. *Nature* **459,** 657–662 (2009).
15. Goffeau, A. *et al.* Life with 6000 Genes. *Science.* **274,** 546–567 (1996).
16. Dietrich, F. S. *et al.* The *Ashbya gossypii* genome as a tool for mapping the ancient *Saccharomyces cerevisiae* genome. *Science* **304,** 304–7 (2004).
17. Souciet, J.-L. *et al.* Comparative genomics of protoploid Saccharomycetaceae. *Genome Res.* **19,** 1696–1709 (2009).
18. Gordon, J. L. *et al.* Evolutionary erosion of yeast sex chromosomes by mating-type switching accidents. *Proc. Natl. Acad. Sci. U. S. A.* **108,** 20024–9 (2011).
19. Ciss, O. H. *et al.* Genome Sequencing of the Plant Pathogen. **4,** 1–8 (2013).
20. Wood, V. *et al.* PomBase: A comprehensive online resource for fission yeast. *Nucleic Acids Res.* **40,** 695–699 (2012).
21. Rhind, N. *et al.* Comparative functional genomics of the fission yeasts. *Science.* **332,** 930–6 (2011).
22. Cissé, O. H., Pagni, M. & Hauser, P. M. De novo assembly of the *Pneumocystis jirovecii* genome from a single bronchoalveolar lavage fluid specimen from a patient. *MBio* **4,** 1–4 (2012).
23. Joint Genome Institute. at <http://http//genome.jgi.doe.gov/>
24. Kämper, J. *et al.* Insights from the genome of the biotrophic fungal plant pathogen *Ustilago maydis*. *Nature* **444,** 97–101 (2006).
25. Loftus, B. J. *et al.* The Genome of the Basidiomycetous Yeast and Human Pathogen *Cryptococcus neoformans*. *Science.* **307,** 1321–1324 (2005).
26. Duplessisa, S. *et al.* Obligate biotrophy features unraveled by the genomic analysis of rust fungi. *Proc. Natl. Acad. Sci. U. S. A.* 1–23 (2011).
27. Stajich, J. E. *et al.* Insights into evolution of multicellular fungi from the assembled

chromosomes of the mushroom *Coprinopsis cinerea* (*Coprinus cinereus*). *Pnas* **107,** 11889–11894 (2010).
28. Martin, F. *et al.* The genome of *Laccaria bicolor* provides insights into mycorrhizal symbiosis. *Nature* **452,** 88–92 (2008).
29. Ma, L. J. *et al.* Genomic analysis of the basal lineage fungus *Rhizopus oryzae* reveals a whole-genome duplication. *PLoS Genet.* **5,** (2009).
30. Eichinger, L. *et al.* The genome of the social amoeba *Dictyostelium discoideum*. *Nature* **435,** 43–57 (2005).
31. King, N. *et al.* The genome of the choanoflagellate *Monosiga brevicollis* and the origin of metazoans. *Nature* **451,** 783–8 (2008).
32. Fairclough, S. R. *et al.* Premetazoan genome evolution and the regulation of cell differentiation in the choanoflagellate *Salpingoeca rosetta*. *Genome Biol.* **14,** R15 (2013).
33. Lander, E. S. *et al.* Initial sequencing and analysis of the human genome. *Nature* **409,** 860–921 (2001).
34. The Arabidopsis Genome Initiative. Analysis of the genome sequence of the flowering plant *Arabidopsis thaliana*. *Nature* **408,** 796–815 (2000).
35. Chen, Y. *et al.* Hydrophobic handoff for direct delivery of peroxisome tail-anchored proteins. *Nat. Commun.* **5,** 5790 (2014).
36. Yuan, P. *et al.* A HEX-1 crystal lattice required for Woronin body function in *Neurospora crassa*. *Nat. Struct. Biol.* **10,** 264–70 (2003).
37. Liu, F. *et al.* Making two organelles from one: Woronin body biogenesis by peroxisomal protein sorting. *J. Cell Biol.* **180,** 325–339 (2008).
38. Ng, S. K., Liu, F., Lai, J., Low, W. & Jedd, G. A tether for Woronin body inheritance is associated with evolutionary variation in organelle positioning. *PLoS Genet.* **5,** e1000521 (2009).
39. Lai, J. *et al.* Intrinsically disordered proteins aggregate at fungal cell-to-cell channels and regulate intercellular connectivity. *Proc. Natl. Acad. Sci.* **109,** 15781–15786 (2012).
40. van Peer, A. F. *et al.* The septal pore cap is an organelle that functions in vegetative growth and mushroom formation of the wood-rot fungus *Schizophyllum commune*. *Environ. Microbiol.* **12,** 833–44 (2010).
41. Leeder, A. C., Jonkers, W., Li, J. & Louise Glass, N. Early colony establishment in *Neurospora crassa* requires a MAP kinase regulatory network. *Genetics* **195,** 883–898 (2013).
42. Aldabbous, M. S. *et al.* The *ham-5*, *rcm-1* and *rco-1* genes regulate hyphal fusion in *Neurospora crassa*. *Microbiology* **156,** 2621–2629 (2010).
43. Fu, C. *et al.* Identification and characterization of genes required for cell-to-cell fusion in *Neurospora crassa*. *Eukaryot. Cell* **10,** 1100–9 (2011).
44. Fleißner, A. *et al.* The *so* locus is required for vegetative cell fusion and postfertilization events in *Neurospora crassa*. *Eukaryot. Cell* **4,** 920–930 (2005).
45. Engh, I. *et al.* The WW domain protein PRO40 is required for fungal fertility and associates with Woronin bodies. *Eukaryot. Cell* **6,** 831–43 (2007).
46. Maddi, A., Dettman, A., Fu, C., Seiler, S. & Free, S. J. WSC-1 and HAM-7 are MAK-1 MAP kinase pathway sensors required for cell wall integrity and hyphal fusion in *Neurospora crassa*. *PLoS One* **7,** e42374 (2012).
47. Masloff, S., Pöggeler, S. & Kück, U. The *pro1(+)* gene from *Sordaria macrospora* encodes a C6 zinc finger transcription factor required for fruiting body development. *Genetics* **152,** 191–9 (1999).
48. Nowrousian, M. *et al.* The novel ER membrane protein PRO41 is essential for sexual development in the filamentous fungus *Sordaria macrospora*. *Mol. Microbiol.* **64,** 923–937 (2007).

49. Lacaze, I., Lalucque, H., Siegmund, U., Silar, P. & Brun, S. Identification of NoxD/Pro41 as the homologue of the p22phox NADPH oxidase subunit in fungi. *Mol. Microbiol.* **95,** 1006–1024 (2015).
50. Ohm, R. a., de Jong, J. F., de Bekker, C., Wösten, H. a B. & Lugones, L. G. Transcription factor genes of Schizophyllum commune involved in regulation of mushroom formation. *Mol. Microbiol.* **81,** 1433–1445 (2011).
51. Horiuchi, J., Silverman, N., Piña, B., Marcus, G. a & Guarente, L. ADA1, a novel component of the ADA/GCN5 complex, has broader effects than GCN5, ADA2, or ADA3. *Mol. Cell. Biol.* **17,** 3220–8 (1997).
52. Malapi-Wight, M., Kim, J. E. & Shim, W. B. The N-terminus region of the putative C2H2 transcription factor Ada1 harbors a species-specific activation motif that regulates asexual reproduction in *Fusarium verticillioides*. *Fungal Genet. Biol.* **62,** 25–33 (2014).
53. Kothe, G. O. & Free, S. J. The isolation and characterization of *nrc-1* and *nrc-2*, two genes encoding protein kinases that control growth and development in *Neurospora crassa*. *Genetics* **149,** 117–130 (1998).
54. Dettmann, A., Heilig, Y., Valerius, O., Ludwig, S. & Seiler, S. Fungal communication requires the MAK-2 pathway elements STE-20 and RAS-2, the NRC-1 adapter STE-50 and the MAP kinase scaffold HAM-5. *PLoS Genet.* **10,** e1004762 (2014).
55. Liu, Y. An Essential Gene for Fruiting Body Initiation in the Basidiomycete *Coprinopsis cinerea* Is Homologous to Bacterial Cyclopropane Fatty Acid Synthase Genes. *Genetics* **172,** 873–884 (2005).
56. Araujo-Palomares, C. L., Richthammer, C., Seiler, S. & Castro-Longoria, E. Functional characterization and cellular dynamics of the CDC-42 - RAC - CDC-24 module in *Neurospora crassa*. *PLoS One* **6,** e27148 (2011).
57. Li, D. A Mitogen-Activated Protein Kinase Pathway Essential for Mating and Contributing to Vegetative Growth in *Neurospora crassa*. *Genetics* **170,** 1091–1104 (2005).
58. Maerz, S. *et al.* The nuclear Dbf2-related kinase COT1 and the mitogen-activated protein kinases MAK1 and MAK2 genetically interact to regulate filamentous growth, hyphal fusion and sexual development in *Neurospora crassa*. *Genetics* **179,** 1313–1325 (2008).
59. Pandey, A., Roca, M. G., Read, N. D. & Glass, N. L. Role of a mitogen-activated protein kinase pathway during conidial germination and hyphal fusion in *Neurospora crassa*. *Eukaryot. Cell* **3,** 348–58 (2004).
60. Park, G., Pan, S. & Borkovich, K. A. Mitogen-activated protein kinase cascade required for regulation of development and secondary metabolism in *Neurospora crassa*. *Eukaryot. Cell* **7,** 2113–2122 (2008).
61. Angelica, M. D. & Fong, Y. NIH Public Access. *October* **141,** 520–529 (2008).
62. Xiang, Q., Rasmussen, C. & Glass, N. L. The *ham-2* locus, encoding a putative transmembrane protein, is required for hyphal fusion in *Neurospora crassa*. *Genetics* **160,** 169–80 (2002).
63. Bloemendal, S. *et al.* A Mutant Defective in Sexual Development Produces Aseptate Ascogonia. *Eukaryot. Cell* **9,** 1856–1866 (2010).
64. Dettmann, A. *et al.* HAM-2 and HAM-3 are central for the assembly of the *Neurospora* STRIPAK complex at the nuclear envelope and regulate nuclear accumulation of the MAP kinase MAK-1 in a MAK-2-dependent manner. *Mol. Microbiol.* **90,** 796–812 (2013).
65. Simonin, A. R., Rasmussen, C. G., Yang, M. & Glass, N. L. Genes encoding a striatin-like protein (ham-3) and a forkhead associated protein (ham-4) are required for hyphal fusion in *Neurospora crassa*. *Fungal Genet. Biol.* **47,** 855–68 (2010).

66. Bloemendal, S. *et al.* A homologue of the human STRIPAK complex controls sexual development in fungi. *Mol. Microbiol.* **84,** 310–23 (2012).
67. Nowrousian, M., Masloff, S., Pöggeler, S. & Kück, U. Cell differentiation during sexual development of the fungus *Sordaria macrospora* requires ATP citrate lyase activity. *Mol. Cell. Biol.* **19,** 450–60 (1999).
68. Dettmann, A. *et al.* HAM-2 and HAM-3 are central for the assembly of the *Neurospora* STRIPAK complex at the nuclear envelope and regulate nuclear accumulation of the MAP kinase MAK-1 in a MAK-2-dependent manner. *Mol. Microbiol.* **90,** 796–812 (2013).
69. Gesing, S., Schindler, D., Fränzel, B., Wolters, D. & Nowrousian, M. The histone chaperone ASF1 is essential for sexual development in the filamentous fungus *Sordaria macrospora*. *Mol. Microbiol.* **84,** 748–765 (2012).
70. Degli-Innocenti, F. & Russo, V. E. Isolation of new white collar mutants of *Neurospora crassa* and studies on their behavior in the blue light-induced formation of protoperithecia. *J. Bacteriol.* **159,** 757–761 (1984).
71. Tsitsigiannis, D. I., Kowieski, T. M., Zarnowski, R. & Keller, N. P. Endogenous lipogenic regulators of spore balance in *Aspergillus nidulans*. *Eukaryot. Cell* **3,** 1398–1411 (2004).
72. López-Berges, M. S. *et al.* The velvet complex governs mycotoxin production and virulence of *Fusarium oxysporum* on plant and mammalian hosts. *Mol. Microbiol.* **87,** 49–65 (2013).
73. Bayram, Ö. S. *et al.* Laea control of velvet family regulatory proteins for light-dependent development and fungal cell-type specificity. *PLoS Genet.* **6,** 1–17 (2010).
74. Ni, M. & Yu, J. H. A novel regulator couples sporogenesis and trehalose biogenesis in *Aspergillus nidulans*. *PLoS One* **2,** e970 (2007).
75. Ahmed, Y. L. *et al.* The Velvet Family of Fungal Regulators Contains a DNA-Binding Domain Structurally Similar to NF-κB. *PLoS Biol.* **11,** e1001750 (2013).
76. Cano-Domínguez, N., Alvarez-Delfín, K., Hansberg, W. & Aguirre, J. NADPH oxidases NOX-1 and NOX-2 require the regulatory subunit NOR-1 to control cell differentiation and growth in *Neurospora crassa*. *Eukaryot. Cell* **7,** 1352–61 (2008).
77. Jiménez-Ortigosa, C. *et al.* Chitin synthases with a myosin motor-like domain control the resistance of *Aspergillus fumigatus* to echinocandins. *Antimicrob. Agents Chemother.* **56,** 6121–6131 (2012).
78. Fajardo-Somera, R. a *et al.* Dissecting the function of the different chitin synthases in vegetative growth and sexual development in *Neurospora crassa*. *Fungal Genet. Biol.* **75,** 30–45 (2015).
79. Zerbino, D. R. & Birney, E. Velvet: Algorithms for de novo short read assembly using de Bruijn graphs. *Genome Res.* **18,** 821–829 (2008).
80. Trapnell, C., Pachter, L. & Salzberg, S. L. TopHat: Discovering splice junctions with RNA-Seq. *Bioinformatics* **25,** 1105–1111 (2009).
81. Trapnell, C. *et al.* Transcript assembly and abundance estimation from RNA-Seq reveals thousands of new transcripts and switching among isoforms. *Nat. Biotechnol.* **28,** 511–515 (2011).
82. Grabherr, M. G. *et al.* Full-length transcriptome assembly from RNA-Seq data without a reference genome. *Nat. Biotechnol.* **29,** 644–52 (2011).
83. Haas, B. J. *et al.* Improving the Arabidopsis genome annotation using maximal transcript alignment assemblies. *Nucleic Acids Res.* **31,** 5654–5666 (2003).
84. Stanke, M. *et al.* AUGUSTUS: *Ab initio* prediction of alternative transcripts. *Nucleic Acids Res.* **34,** 435–439 (2006).
85. Korf, I. Gene finding in novel genomes. *BMC Bioinformatics* **5,** 59 (2004).
86. Ter-Hovhannisyan, V., Lomsadze, A., Chernoff, Y. O. & Borodovsky, M. Gene

prediction in novel fungal genomes using an *ab initio* algorithm with unsupervised training. *Genome Res.* **18,** 1979–90 (2008).
87. Cantarel, B. L. *et al.* MAKER: An easy-to-use annotation pipeline designed for emerging model organism genomes. *Genome Res.* **18,** 188–196 (2008).
88. Wallace, I. M., O'Sullivan, O., Higgins, D. G. & Notredame, C. M-Coffee: Combining multiple sequence alignment methods with T-Coffee. *Nucleic Acids Res.* **34,** 1692–1699 (2006).
89. Capella-Gutiérrez, S., Silla-Martínez, J. M. & Gabaldón, T. trimAl: A tool for automated alignment trimming in large-scale phylogenetic analyses. *Bioinformatics* **25,** 1972–1973 (2009).
90. Rokas, A. Phylogenetic analysis of protein sequence data using the Randomized Axelerated Maximum Likelihood (RAXML) Program. *Curr. Protoc. Mol. Biol.* **Chapter 19,** Unit19.11 (2011).
91. Le, S. Q. & Gascuel, O. An improved general amino acid replacement matrix. *Mol. Biol. Evol.* **25,** 1307–1320 (2008).
92. Li, L., Stoeckert, C. J. J. & Roos, D. S. OrthoMCL: Identification of Ortholog Groups for Eukaryotic Genomes. *Genome Res.* **13,** 2178–2189 (2003).
93. Pearson, W. R. Finding protein and nucleotide similarities with FASTA. *Curr. Protoc. Bioinforma.* **Chapter 3,** Unit3.9 (2016).
94. Csurös, M. Count: Evolutionary analysis of phylogenetic profiles with parsimony and likelihood. *Bioinformatics* **26,** 1910–1912 (2010).
95. Han, M. V., Thomas, G. W. C., Lugo-Martinez, J. & Hahn, M. W. Estimating gene gain and loss rates in the presence of error in genome assembly and annotation using CAFE 3. *Mol. Biol. Evol.* **30,** 1987–1997 (2013).
96. Edgar, R. C. MUSCLE: Multiple sequence alignment with high accuracy and high throughput. *Nucleic Acids Res.* **32,** 1792–1797 (2004).
97. Guindon, S. *et al.* New algorithms and methods to estimate maximum-likelihood phylogenies: Assessing the performance of PhyML 3.0. *Syst. Biol.* **59,** 307–321 (2010).
